# Infection threat shapes our social instincts

**DOI:** 10.1007/s00265-021-02975-9

**Published:** 2021-02-10

**Authors:** Peter Kramer, Paola Bressan

**Affiliations:** Dipartimento di Psicologia Generale, Università di Padova, Via Venezia 8, 35131 Padua, Italy

**Keywords:** Behavioral immune system, Infectious disease, Ingroup/outgroup, Disgust, Oxytocin, Public health

## Abstract

We social animals must balance the need to avoid infections with the need to interact with conspecifics. To that end we have evolved, alongside our physiological immune system, a suite of behaviors devised to deal with potentially contagious individuals. Focusing mostly on humans, the current review describes the design and biological innards of this behavioral immune system, laying out how infection threat shapes sociality and sociality shapes infection threat. The paper shows how the danger of contagion is detected and posted to the brain; how it affects individuals’ mate choice and sex life; why it strengthens ties within groups but severs those between them, leading to hostility toward anyone who looks, smells, or behaves unusually; and how it permeates the foundation of our moral and political views. This system was already in place when agriculture and animal domestication set off a massive increase in our population density, personal connections, and interaction with other species, amplifying enormously the spread of disease. Alas, pandemics such as COVID-19 not only are a disaster for public health, but, by rousing millions of behavioral immune systems, could prove a threat to harmonious cohabitation too.

*L’enfer c’est les autres.*

(*Hell is other people*)

- Jean-Paul Sartre, Huis Clos, 1943

## Safety lies in solitude

We social animals all face an irresolvable dilemma: we depend upon other individuals to survive, but others carry germs that can kill us. In our species, many such germs are passed around just by talking or breathing; coughs and sneezes conveniently wrap them inside a moist gas cloud which travels up to 8 meters, producing residues that may hang in the air for hours (Bourouiba 2020). Viruses, bacteria, and all manner of microorganisms kill, every year, 15 million people directly and—by setting off all sorts of health consequences and complications—several additional millions indirectly (Morens et al. 2004). They appear to meddle even in human diseases that are commonly deemed nontransmissible (Cochran et al. 2000), like cancer (Palmer et al. 2018), stroke and heart disease (Li et al. 2020), autoimmune conditions such as celiac disease and multiple sclerosis (Bar-Or et al. 2020; Jiang et al. 2020), and mental and neurodegenerative disorders like schizophrenia and Alzheimer’s (Kramer and Bressan 2015; Osorio et al. 2019). Parasites—here broadly defined to include all pathogens—have indeed been the main driver of human evolution (Fumagalli et al. 2011), and a rather frenetic one at that: a gene that steps up our defenses against them (CEACAM3: Adrian et al. 2019; see also Corona et al. 2018) is among those that are now evolving the fastest.

**Fig. 1:**
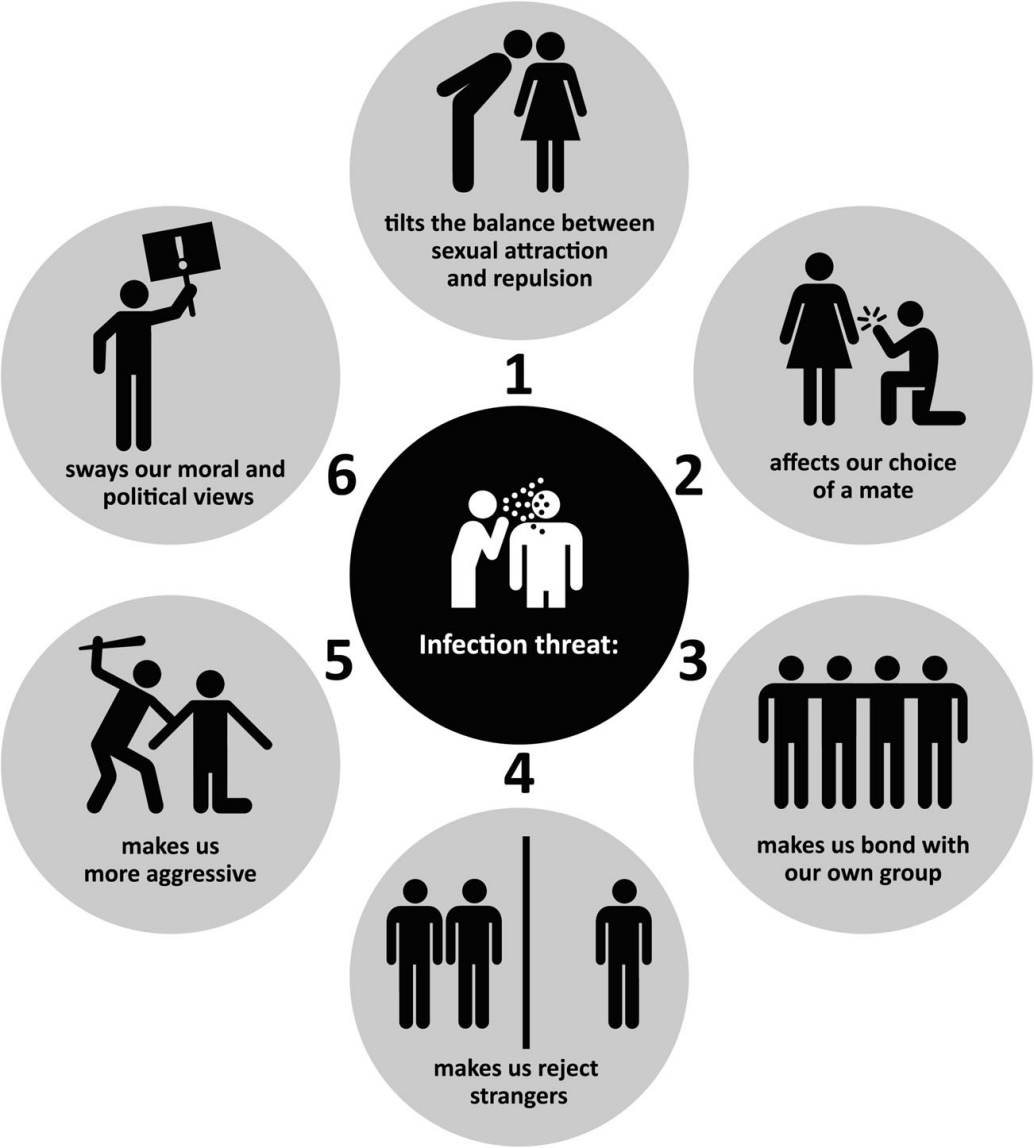
The many ways in which infection threat shapes social behavior. (Icons: 0—public domain, adapted; 1—Yeong Rong Kim, from the Noun Project; 2—Luis Prado, from the Noun Project; 3—Andrew Doane, from the Noun Project; 4—Anton Håkanson, from the Noun Project, adapted; 5—public domain; 6—public domain, adapted.) Image copyright by Paola Bressan

In this paper we offer a broad perspective on how and why infection threat shapes social behavior (Fig. 1), and how social behavior, in turn, alters infection threat. Our review updates previous ones on this topic, but differs from them in two major ways. First, none of it is based on comparisons between geographical regions. These comparisons suggest that people who live in places with larger parasite threats tend to be more introverted, closed-minded, intolerant, collectivistic (rather than individualistic), religious, traditionalistic, conservative, ethnocentric, xenophobic, and violent (Thornhill and Fincher 2014). Such associations are consistent with those discussed here. Yet because, either potentially or in actual fact, these studies suffer from methodological issues which are difficult to overcome (Bromham et al. 2018), we do not consider them. Our review is, instead, placed on a stronger footing, relying as it does on controlled experiments on humans and other social animals, comparisons between individuals rather than regions, and analyses of historical developments within regions. The second way in which this review differs from previous ones is that it is broader: it integrates findings from fields that are regarded as independent from one another, but that have partly overlapping subject matters, are mutually informative, and can help constrain one another’s theories or inspire their development.

## Detecting infection

We all start accumulating both temporary and lifelong infections as soon as we are conceived, a string of tribulations that never stops (Kramer and Bressan 2015). Clearly, a well-honed immune system is far from a guarantee that we will stay safe from pathogens. Along with this immune system, which for want of a better term we shall call “physiological,” we have thus also evolved a behavioral one—a set of mechanisms (whose underpinnings, of course, are still physiological) that prompt us to *behave* so as to avoid infections (Murray and Schaller 2016; Lieberman et al. 2018; Sarabian et al. 2018).

The first order of business is to collect information about who might be contagious. If someone else’s germs have already invaded us, our brain should of course urgently be informed about this too—a job which the physiological immune system takes on itself. Indeed, the physiological and behavioral immune systems are not independent but talk to each other a great deal: one may see them as complementary aspects of a single, integrated immune system (Clark and Fessler 2014; Gangestad and Grebe 2014).

### Follow your nose

People’s physiological immune system leaves behind clues about its strengths and weaknesses and, most pressingly, about whether it happens to be fighting an infection. On their surface, cells display a sample of their protein content. If such proteins belong to the cell itself, the cell is left untouched. If not, suspicion falls on parasite invasion: the cell is then attacked (with the help of immune cells such as white blood cells), killed, and subsequently replaced. The genes that coordinate the production of the proteins that cells expose on their outside are collectively known as the MHC (major histocompatibility complex). The greater the diversity within an individual’s MHC, the more information about potentially infected cells this person’s immune system has—and the better an immune response it can mount against them (Howard 1991).

The MHC marks the scent of urine (both in mice and in humans: Eggert et al. 1999) and is quite likely to affect the odor of one’s breath, sweat, and genitals as well (Wobst 1999; Aksenov et al. 2012; Grogan 2019). It can also alter the menagerie of microbes individuals carry around (Khan et al. 2019), and thus the aromatic gases such microbes give off (Rudden et al. 2020). Together, all these odors form a sort of olfactory fingerprint. By smelling it, people appear able to (unconsciously) assess some characteristics of others’ immune system (Milinski et al. 2013).

Contrary to common belief, humans (women especially: Sorokowski et al. 2019) are graced with a sense of smell that for some odors is as good as that of dogs and mice, or better (McGann 2017). Such a sense of smell can help us find out who is a family member or a suitable partner; who is young or old; who does or does not share our ethnicity; who is happy, afraid, or disgusted; and who harbors aggressive intentions (see Marxer-Tobler and Pineda 2012; Bressan and Kramer 2015; Mutic et al. 2016; Winternitz et al. 2017). Likewise, we can smell people who are infected (Shirasu and Touhara 2011; Moshkin et al. 2012) or even just pseudo-infected—that is, who are carrying bacterial parts that activate the immune system without causing an actual infection (Olsson et al. 2014; Regenbogen et al. 2017). This subtle distinction is instructive because, during an infection, changes in our body odor could in principle be due either to the invading parasites or to our response to them; but during a pseudo-infection, only the latter option remains.

### Follow your eyes

Infection and pseudo-infection not only change one’s smell (Moshkin et al. 2012), but also redden the eyes (Axelsson et al. 2018), pale the lips and the skin, and alter facial expression (Sarolidou et al. 2019) and gait (Sundelin et al. 2015; Lasselin et al. 2020). People like a portrait less when the depicted person has been photographed after getting pseudo-infected than after getting a placebo injection with saline, in particular if the photo is accompanied by that person’s armpit odor (Regenbogen et al. 2017; Sarolidou et al. 2020).

Entirely harmless pictures or descriptions of dirty toilets, infected eyes, and suchlike not only provoke disgust (Curtis et al. 2004; Curtis and de Barra 2018) but are enough to raise body temperature and sensitivity to pain (Stevenson et al. 2012; Oaten et al. 2015; Stevenson et al. 2015; see also Cruz-Albarran et al. 2017). They also launch a stronger immune reaction than do photos of a man pointing a gun (Schaller et al. 2010; see also Stevenson et al. 2012). Compared to others, pictures, videos, or descriptions of health hazards attract indeed more attention (Chapman et al. 2013; van Hooff et al. 2013), are remembered better (Chapman et al. 2013; Gretz and Huff 2019), make us want more personal space (Park 2015), and promote intentions to buy and use condoms (Tybur et al. 2011). Such physiological and behavioral reactions, which are larger in people who have encountered infectious disease more often, have a point: people who get disgusted sooner and are more afraid of contamination contract fewer infections (Stevenson et al. 2009).

### Follow your cytokines

In a way, our immune system is a sense just as much as are our vision and smell (Kipnis 2018). While vision and smell convey to the brain information about the outer world, the immune system does the same about our inner one—a bit like proprioception, the sense of one’s own posture and movement. If we are being invaded, our immune system dispatches white blood cells in a counterattack known as inflammation. These cells release proinflammatory signaling molecules called cytokines, like interferon gamma and interleukins 1 and 17, which call reinforcements to the scene. Once mission is accomplished, white blood cells release instead anti-inflammatory molecules such as interleukins 4 and 10, alerting the immune system that it is time to switch the inflammation off (Kipnis 2018; Gassen and Hill 2019). Unlike the white blood cells themselves, cytokines can pass the blood-brain barrier and keep the brain posted about the developments; virtually all neuron types carry receptors that can be activated by these. In fact, the immune and nervous systems interact quite extensively (Kipnis 2018; Gassen and Hill 2019).

It is the bone marrow that produces white blood cells; and mice who receive a bone marrow transplant from mice with high levels of (proinflammatory) interleukin 6 become more socially withdrawn, and seemingly depressed, in response to a stress like being defeated by a large aggressive mouse, than those who get the transplant from normal mice (Hodes et al. 2014; see also Hodes et al. 2016; Takahashi et al. 2018; Wittenberg et al. 2020). Note that becoming unsociable, hence keeping to oneself, helps ward off further infection. Indeed, after watching a slide show about germs, people who feel particularly vulnerable to disease tend to become more introverted, and less open to new experiences, than people who have watched a slide show about something else (Mortensen et al. 2010).

Mice that, because of a genetic mutation, are unable to produce antibodies spend less time exploring one another (but not less time investigating new objects) than do normal mice. Yet their social behavior becomes normal as soon as immunity is restored (Filiano et al. 2016). In several species, the connection between social behavior and response against pathogens appears to be mediated by (proinflammatory) interferon gamma. This molecule’s signaling activity is upregulated in organisms that live together, among which the risk of infection is higher, and downregulated in animals that are socially isolated, among which it is lower (Filiano et al. 2016). In humans, one variant of the gene that regulates the production of interferon gamma renders its bearers more susceptible to infections such as malaria and tuberculosis: revealingly, people who carry this variant appear more inclined to avoid harm, and are possibly less extrovert and willing to explore (MacMurray et al. 2014; see also Luchetti et al. 2014; Napolioni et al. 2014).

## The benefit of procreation against the cost of infection

Members of a social species can hardly avoid all of their conspecifics all of the time, especially if they rely on sex for reproduction; a balance must be struck between engaging with others and coping with the peril of contagion (Sawada 2018; Tybur et al. 2020).

### Masculine mates, feminine mates, or something in between

One way to deal with the risks and consequences of infection is to accept the risks and mitigate the consequences—at least those that will weigh upon one’s offspring. Naturally, a solid plan of action is to produce such offspring with certain partners and not others.

Men’s masculinity (broad jaws, thick brows) and women’s femininity (large eyes, full lips) are viewed as attractive features—though this is much clearer for femininity, an unmitigated asset (Perrett et al. 1998; Rhodes 2006), than for masculinity, which involves costs too (Stephen et al. 2012; Lee et al. 2015; Foo et al. 2017). Among such costs are, for example, being a more aggressive and less faithful husband (Booth and Dabbs 1993). Masculinity and femininity develop during adolescence in response to sex hormones such as testosterone and estrogen, whose production depends on mitochondria—former bacteria now residing inside our cells, best known for turning the food we eat into the energy we live on (Kramer and Bressan 2018, 2019).

Mitochondria are involved in the workings of the immune system too (Bordt et al. 2019); and how expertly the immune system of a teenager runs predicts his masculinity, or her femininity, in adulthood (Foo et al. 2020). Masculinity and femininity thus come forth as honest signals of immune function at an important stage of development. Because the need to produce particularly vigorous children rises with infection threat, so does the importance of securing a physically attractive mate—as opposed to one with traits such as domestic skills or financial prospects, which are pleasing but not as helpful in making one’s offspring resistant to parasites (Gangestad et al. 2006). There is indeed evidence that infection threat is associated with a preference for masculine men and feminine women (Lee and Zietsch 2011; Little et al. 2011; de Barra et al. 2013; Jones et al. 2013; see also Lee et al. 2015). Yet interestingly, a man’s *current* healthiness—as advertized, in particular, by a more yellow/orange skin color, reflecting a diet richer in fruit and vegetables’ carotenoids—affects his attractiveness more than does the masculinity of his facial traits (Scott et al. 2010; Whitehead et al. 2012).

### Sexual attraction, sexual repulsion, or something in between

A very different way to go about the problem is to balance the cost of risking an infection and the benefit of producing offspring, and act accordingly. Indeed, whether we engage in sex or not depends partly on whether we are disgusted or inclined to feel disgusted. Images of garbage, feces, or corpses (Andrews et al. 2015; Fleischman et al. 2015), or a mixture of the odors associated with smelly feet, rotten garlic, and skunks (Borg et al. 2019), reduce sexual arousal while one is watching erotic films or pictures. This automatic response is bound to decrease one’s willingness to have sex, and thus helps to avoid infection. At least in men, the converse is also true: being sexually aroused reduces disgust and fear of sexually transmitted infections, which of course clears the way for reproduction (Oaten et al. 2019). In women—who tread more carefully in these matters—the evidence is mixed (Fleischman et al. 2015). Men and women who are more interested in casual sex are less prone to sexual disgust, in line with the idea that this is calibrated by one’s mating strategy (Al-Shawaf et al. 2015; O’Shea et al. 2019). Possibly in the attempt to compensate for a larger risk of contagion due to increased potential for close contact with a new person, the immune system of women in love appears upregulated (Murray et al. 2019a). And when women become mothers, the tension between the need to look after the next generation and the need to avoid infection shows up in their attitude toward baby feces too: they find the smell of their own baby’s excrements less repulsive than that of other babies’, even when they do not know which baby produced which (Case et al. 2006).

Although they have stronger immune responses and suffer less from infections, women are disgusted much more easily than men (for a meta-analysis, see Sparks et al. 2018; for findings in rodents, see Kavaliers et al. 2020). Researchers have come up with a number of possible explanations (Al-Shawaf et al. 2018). In the course of evolution, for example, increased disgust may have lowered women’s risks of infecting themselves and their offspring while preparing food and caring for children—but, at the same time, may have stood in the way of typically male bloody practices like hunting and warfare. Another point is that women benefit less from casual sex than men do and contract sexually transmitted infections more easily; such infections can then be passed on to children, particularly when they are still in the womb. Indeed, it is especially with regard to sexual matters that women are more easily disgusted than men (Sparks et al. 2018). A side effect of the difference in disgust between the sexes appears to be that women suffer more than men from disgust-related mental problems like obsessive-compulsive disorder, eating disorders, and animal phobias (Al-Shawaf et al. 2018; Amoroso et al. 2020; see also Viol et al. 2019).

Women are more liable to contract an infection in the part of the cycle that follows ovulation (the luteal phase), when the body prepares itself for a possible pregnancy, than during the phase that leads up to it (the follicular phase: Fessler 2002). During the luteal phase, as during the first trimester of pregnancy, the immune system is damped down to prevent it from attacking any fertilized cells that might be starting to develop; after all, just like parasites do, such cells happen to carry foreign genetic material. It has indeed been reported that, among women who are currently fighting an infection, being in the luteal rather than the follicular phase increases pathogen disgust (Milkowska et al. 2019; see also Pilarczyk et al. 2019). Pathogen disgust is also higher in women who are in the first three months of pregnancy (Fessler et al. 2005).

### Sexual attraction, sexual repulsion: biological bases

Prominent in orchestrating the tension between approach and avoidance in many different species, ranging from fish and amphibians to birds and mammals, is the hormone and neurotransmitter oxytocin (Wacker and Ludwig 2012; see also Duque-Wilckens et al. 2020). Oxytocin stimulates delivery, lactation, and maternal behavior, and has been celebrated for its positive effects on trust and social bonding (Kosfeld et al. 2005). Recent evidence, however, shows that it encourages not only *pro*social, but also *a*social and—as we will discuss later—even *anti*social behavior (De Dreu and Kret 2016). In a further twist, oxytocin, its sister vasopressin, and one of the brain areas with which they extensively interact—the amygdala—all turn out to be involved, at least in rodents, in the processing of the odors of others (Wacker and Ludwig 2012) and in the regulation of both the physiological and behavioral immune systems (physiological: Li et al. 2017; Bordt et al. 2019; behavioral: Choleris et al. 2012; Kavaliers and Choleris 2018; Kavaliers et al. 2020).

In the fertile hours of their 4–5 days estrous cycle, female mice prefer the odor of *unfamiliar* males (Kavaliers et al. 2003b), presumably because strangers are less likely to be kin and thus more suitable as sex partners (Sherborne et al. 2007). Yet such unfamiliar males could carry unfamiliar parasites to which one’s immune system has not yet adapted and that may prove particularly dangerous (McNeill 1976; Fincher and Thornhill 2012; Bressan 2020). Mice seem well tuned in to this possibility (Kavaliers et al. 2014), and fertile females decrease their preference for unfamiliar males if they have previously smelled an infected male, and are keener to avoid infected males if they have previously smelled an unfamiliar male. If their oxytocin gene is deleted, females become unable to respond to males’ familiarity and infectiousness but are still repelled by the smell of a cat (Kavaliers et al. 2003a): oxytocin thus instills avoidance of infection in particular, not of danger in general.

Fertile females also like a male’s odor best if it is accompanied by the odor of another fertile female. That is, just like human females, they prefer popular males (Kavaliers et al. 2017). This preference does not show up if the female who provides the accompanying odor is infected. Evidently, even an indirect risk of contagion reduces the appeal of an otherwise attractive potential mate. Yet again, neither the preference for males associated with an uninfected female nor the aversion to those associated with an infected one materialize without a functioning oxytocin receptor (Kavaliers et al. 2019).

Notably, oxytocin and vasopressin interact with sex hormones like estrogen and testosterone (Gabor et al. 2012; Li et al. 2017), which too have been implicated in balancing approach and avoidance. Taking certain estrogen receptors out of service has repercussions akin to sabotaging the oxytocin receptor (Choleris et al. 2012; Kavaliers and Choleris 2018).

In women in the luteal phase or the first trimester of pregnancy, the suppression of immunity is brought about by rising levels of the sex hormone progesterone (Fessler 2002). It thus makes intuitive sense that women’s worries about contamination, and disgust for photographs associated with it, increase with progesterone levels in saliva (Fleischman and Fessler 2011). In apparent contradiction, a large study has failed to show that pathogen disgust in women is affected by the natural variation of any sex or stress hormone—be it progesterone, estrogen, testosterone, or cortisol (Jones et al. 2018). Note, however, that this study did not use photographs and measured disgust variations by repeatedly presenting (typically 5–10 times) the very same questionnaire to the very same women. It is therefore hard to exclude the possibility that, in responding to later questionnaires, participants were influenced by what they had responded to earlier ones, a potential glitch that could have obscured any underlying fluctuation in disgust. Incidentally, an injection of progesterone does increase “disgust”—avoidance of infected males—in female mice, as shown by Bressan and Kramer’s (2021) analysis of Kavaliers et al.’s (2021) data.

## The benefit of social interaction against the cost of infection

Throughout our evolutionary history, we have largely been incapable of dealing with germs directly. Instead, we have adapted to deal with the creatures who are likeliest to infect us: our fellow humans—and some more than others.

### Us versus them

The more easily we feel disgusted, the more collectivistic (as opposed to individualistic) we are inclined to be (Clay et al. 2012); that is, the more we view our identity as integrated with that of a group, rather than independent from it. Collectivism is characterized by strong family and community bonds, which might help recruit support in times of hardship and illness; evidence for support to group members in need is plentiful not only in humans (Fincher and Thornhill 2012), but in animals too (Hart 2011). And the more disgusted or vulnerable to disease we feel, the more we conform to our community’s norms, traditions, and habits—and are disturbed by their violation (Murray and Schaller 2012; Wu and Chang 2012; Tybur et al. 2016; Murray et al. 2019b). It is no accident that many such practices—above all those regarding food preparation and personal hygiene—help prevent infection or deal with its consequences (McNeill 1976; Fabrega 1997; Schaller and Neuberg 2008). Note, however, that this cannot be the whole story, as one can find at least as many rituals that hasten the spread of disease instead—think of religious gatherings, ceremonial baths of different people in the same water, or the Holy Communion (see also McNeill 1976).

Misjudging a contagious individual as healthy tends to have worse consequences than misjudging a healthy one as contagious. The behavioral immune system should thus have evolved to err on the side of caution (Haselton and Nettle 2006), a propensity which ought to increase further when one feels especially vulnerable to disease (Sarolidou et al. 2020; see also Oaten et al. 2017). Indeed, the greater the infection threat, the more animals (Hart 2011) and humans (Murray and Schaller 2016) engage in conducts that distance them from one another (see also Aarøe et al. 2016). It is primarily people who seem ill that we steer clear of (Crandall and Moriarty 1995). Yet, possibly because parasites make people look or behave unusually, our hypervigilant concern about pathogens leads us to perceive *any* abnormal appearance or behavior as a possible sign of infection (Kurzban and Leary 2001; Nussinson et al. 2018). So the circle of people we tend to avoid, in particular if we feel vulnerable to infection, extends to those who are disabled (Park et al. 2003), overweight (Park et al. 2007; Tapp et al. 2020), or mentally ill (Lund 2014); to those who are attracted to people of the same sex (Kiss et al. 2020); and to those who have an unfamiliar ethnicity (Faulkner et al. 2004). Likewise, primate groups have been observed to keep newcomers semi-quarantined for weeks or months at the periphery of the group before accepting them in their midst (Hart 2011). This has been argued to ensure relatively harmless, low-level exposure to the newcomer’s pathogens and thus the opportunity to develop immunity to them (Hart 2011).

Individuals who come from elsewhere are more likely than community members to carry germs which one has not been previously exposed and built up immunity to (Diamond 1997; Thornhill and Fincher 2014). It is indeed telling that the less familiar a face is, the sicker it looks, and unfamiliar faces with an inauspicious rash on them look considerably sicker than familiar ones with the same rash (shown by Bressan 2020’s analysis of van Leeuwen and Petersen 2018’s data). It is also telling that we perceive apples or tomatoes as healthier when we are told they are local rather than foreign (Gineikiene et al. 2016); that disgusting statements alter heart rate, induce automatic avoidance reactions, and evoke feelings of disgust more strongly when made by unfamiliar than by familiar individuals (Peng et al. 2013; Reicher et al. 2016); that a foul smell seems less pleasant when it emanates from a stranger (Stevenson and Repacholi 2005); that the inclination to be disgusted by body odor goes together with prejudice against immigrants (Zakrzewska et al. 2019); and that the more easily disgusted we are, the less similar we feel to people we are unacquainted with (Mentser and Nussinson 2020) and the more distant from our own a foreign accent sounds to us (Reid et al. 2012).

Strangers may also be unaware of the norms and traditions of societies other than their own and so especially likely to violate them (Thornhill and Fincher 2014). Inasmuch as these local norms and traditions protect from local parasites, then, transgressors increase the risk of infection; one more reason why a stronger propensity to be disgusted increases our disapproval of immigrants who do not adjust themselves to our norms (Karinen et al. 2019)—or of any other offenders, for that matter (Tybur et al. 2016).

Even though nearly our entire evolution took place before we found out about germs, the idea of contagion shapes folk beliefs nonetheless. It is revelatory that the sooner we feel disgusted, the more we are prone to believe in evil spirits which can take possession of us, most notably if we get too close to those who are possessed already (Bastian et al. 2019). Similar beliefs form part of many organized religions and may involve angels, demons, or the devil. In fact, the belief in good and evil spirits goes hand in hand with religiosity and with conservatism, a political ideology in which religiosity plays a relatively large role (Bastian et al. 2015). Religiosity and conservatism are, in turn, associated with collectivism (Cukur et al. 2004). The belief in good and evil spirits is also tied to aversion toward suspicious strangers (Bastian et al. 2015). Completing the circle, watching disgusting images leads to greater fear of sin (Stewart et al. 2020); the proclivity to feel disgusted is associated with fear of sin and fear of God (Stewart et al. 2020); and we tend to view people who do not share our faith—as is more likely the case if they are strangers—as less kind and righteous than those who do (Widman 2009).

### Us versus them: biological bases

If our behavioral immune system rests on the same biology as it does in mice, one would expect our parasite avoidance to be affected a great deal by hormones like oxytocin, vasopressin, estrogen, and testosterone. Such hormones might act as intermediaries between infection threat and those adaptive responses that strengthen our ties to familiar people and sever those to unfamiliar ones.

Although we are unaware of direct tests of this point, infection threat and oxytocin do appear to guide our behavior in parallel ways. Oxytocin, unlike placebo, once sprayed into the nose (Quintana et al. 2018) proceeds to increase people’s disposition to share information with randomly assigned group members (De Wilde et al. 2017), learn from them (Xu et al. 2019), collaborate with them (De Dreu et al. 2010), rate the attractiveness of arbitrary symbols in the same way they do (Stallen et al. 2012), comply with their obviously wrong opinions (Edelson et al. 2015; see also Huang et al. 2014), and lie for their benefit and copy their dishonest behavior (Radke and de Bruijn 2012; Shalvi and De Dreu 2014; see also Aydogan 2017). Some such behaviors, like rating symbols as one’s group does and conforming with group members’ viewpoints, can be interpreted as forms of collectivisms. Recall that collectivism is related to conservatism and religiosity, and these, in turn, to spiritual beliefs: tellingly, self-reported spirituality is associated with oxytocin levels in saliva (Holbrook et al. 2015) and increases if oxytocin is sprayed into the nostrils (Van Cappellen et al. 2016).

In an influential study conducted in the Netherlands (De Dreu et al. 2011), men who had sniffed a dose of either oxytocin or placebo were asked whether they would kill a person with a particular name if this could save five nameless ones. Those who had taken oxytocin turned out to be more willing to sacrifice a person with a German or Arab name than one with a Dutch name. Thus, at least in (Dutch) men, oxytocin appears to inspire favoritism toward one’s own group at the expense of others. Adding volatility to inequity, oxytocin also worsens envy and gloating (Shamay-Tsoory et al. 2009) and can instigate aggressive reactions to provocation (Pfundmair et al. 2018; Romney et al. 2019). It does more than merely promote defensive aggression, too: in a game with three attackers and three defenders, the members of the group who had taken oxytocin rather than placebo coordinated better their attacks (Zhang 2019).

### From infection to aggression

Infection and the immune system’s defense against it, inflammation, are associated with aggressive behavior just like oxytocin is (Takahashi et al. 2018). An upregulation of inflammation may well serve to prepare the body for the increased infection risk any aggressive contact involves (Granger et al. 2000). Yet the association might imply a causal arrow in the opposite direction and be viewed as a component of the behavioral immune system. That is, when one is already fighting an infection and is thus particularly vulnerable to everyone else’s germs, but walking away is difficult, an aggressive demeanor could help reduce the risk of further infection by creating a distance from others.

To some degree, aggression is regulated by the signaling molecules that white blood cells dispatch to inform the brain about ongoing immune activity. Depending on which cytokine is injected into which region of the brain, defensive rage can be dialed either up or down in cats (Zalcman and Siegel 2006; see also Bhatt et al. 2008). Blood levels of the proinflammatory TNF (tumor necrosis factor) and interleukins 1, 6, and 8 are more elevated in individuals who score higher on questionnaires that measure aggression (Suarez et al. 2002, 2004; Marsland et al. 2008)—and levels of interleukin 6 in patients with frequent bouts of rage (as opposed to other psychiatric patients or healthy people: Coccaro et al. 2014). Interleukin 1 also rises in rugby players who are about to start their game (Pesce et al. 2013).

Indicating that this evidence is much more than a bunch of correlations, toning down inflammation diminishes aggression. For example, taking supplements of omega-3, a fatty acid abundant in fish such as sardines and mackerels, reduces aggression in both children and adults (for a meta-analysis across 40 studies, see Gajos and Beaver 2016; see also Bègue et al. 2018). A possible explanation is that omega-3 harms viruses and ruptures the membranes of pathogenic microbes like salmonella and *E. coli*, but not those of beneficial ones like lactobacilli (Alcock et al. 2012); in so doing, it reduces the need for an immune defense against nasty germs in the gut and thus curtails inflammation. The abatement of inflammation, in turn, alters the production of an assortment of neurotransmitters (Takahashi et al. 2018). Higher blood levels of omega-3 go together with less antisocial behavior and callousness in teenagers with attention-deficit/hyperactivity disorder (Gow et al. 2013) and less irritability and anger in patients undergoing immunotherapy (Lotrich et al. 2013). Inmates with higher levels of omega-3 are less aggressive and hostile (Meyer et al. 2015), and those who are given omega-3 supplements break prison rules less often (Gesch et al. 2002; Zaalberg et al. 2010). Conversely, poor nutrition—too few fruits and vegetables, too many industrial snacks and sweets—increases aggression in children and is associated with bullying in teenagers (Jackson 2016; Jackson et al. 2017), with a better diet predicting better behavior in one co-twin than in the other (Jackson 2016).

## Being liberal or conservative in a world of pathogens

Parasites affect not only how we behave but also our ideas about how we (and others, especially those in public office) *ought* to behave. Indeed, the fear of infection turns out to imbue the very basis for such judgments: morality itself.

### Infection threat sways our moral values

Behaviors that breach our ethical standards without exposing us to infection, such as lying, cheating, or stealing, not only appear objectionable but can disgust us as well (Tybur et al. 2009; Lieberman et al. 2018; Stevenson et al. 2019). One possible explanation for this “moral” disgust is that people who break norms that do not protect against pathogens appear more likely to break norms that do, too. Another is that, during evolution, the function of disgust broadened to distance ourselves not just from individuals whose infractions raise our risk of disease, but also from those whose transgressions are harmful in other ways (Tybur et al. 2009). Immoral behavior seems indeed to prompt us to become more easily disgusted, as it amplifies our sensitivity to odors and tastes (Skarlicki et al. 2013).

Violations of society’s norms tend to feel immoral, and morals are typically thought of as universal and abstract. What we actually consider moral, however, is neither universal nor abstract. It depends on our genes and hormones (Fumagalli and Priori 2012; Bernhard et al. 2016), on whether the matter is important to our personal survival (Fessler et al. 2015), and on whether local authorities endorse or condemn it (Fessler et al. 2015). It can also be swayed by a spray of oxytocin in our nose or an electromagnet on our scalp (Shalvi and De Dreu 2014; Kuehne et al. 2015; Aydogan 2017). Empathy, sympathy, reciprocity, reconciliation, self-restraint, and self-sacrifice for unrelated individuals are all observed in nonhuman species too (de Waal 1996; de Waal and Preston 2017). In fact, it has been argued that what we consider moral is purely a product of evolution and may be embodied, that is, associated with concrete bodily sensations such as feeling dirty or clean. This means that not only do moral transgressions provoke disgust (Tybur et al. 2013; Vicario et al. 2017; Giner-Sorolla et al. 2018), but the morals themselves may be based on disgusting or wholesome experiences (Chapman and Anderson 2013). For example, we may find a murder immoral not on account of any lofty principles, but because—in addition to vicariously undergoing the pain of the victim or the victim’s family—we feel disgusted about the bloody mess we imagine this act could produce.

From this perspective, it is understandable that after she had manipulated her husband into stabbing the king to death and she herself had returned the bloodstained daggers to the crime scene, Shakespeare’s Lady Macbeth sought to wash her sin away by frantically scrubbing her hands. A high-profile study found that, after considering unethical (as opposed to ethical) events, participants expressed greater desire for cleansing products and were more likely to prefer an antiseptic wipe to a pencil as a parting gift (Zhong and Liljenquist 2006). Overall, attempts to replicate this Macbeth effect have produced rather mixed results (Earp et al. 2014; Siev et al. 2018), and the same goes for related studies (for debate, see for example Cesario 2014; Huang 2014; Klatzky and Creswell 2014; Tang et al. 2017). Keep in mind, however, that the interpretation of “failed” replications is not always straightforward either (e.g., Bressan 2019; Bryan et al. 2019; Gangestad et al. 2019).

Recently, the Macbeth effect has been replicated twice using better methodology (a within- rather than between-subjects design and the addition of physiological measures). The participants to one of these two studies, while stretched out in an fMRI brain scanner, were presented first with an imaginary scenario in which they had to either lie or tell the truth, and then with the picture of a cleansing or noncleansing product whose desirability they had to rate (Denke et al. 2016). Products such as toothpaste or mouthwash, but not glue or batteries, looked more desirable after the lies than after the truths. Brain scans revealed that this Macbeth effect engaged an area dedicated to handling sensory experiences and planning actions, not to abstract moral thinking. The other study showed a preference for mouthwash products after spoken lies (but not truths), which came with activation of the mouth-related area of the brain, and a preference for handwash products after written lies (but not truths), which came with activation of the hand-related area (Schaefer et al. 2015; see also Schaefer 2019).

Indeed, after we have read about a disease threat, cleansing our hands or feeling protected by vaccination decrease our prejudice toward immigrants (Huang et al. 2011; see also Golec de Zavala et al. 2014). Conversely, the more we feel disgusted—either here and now or in general—the more inclined we are to moralize, condemn, and dole out harsh penalties as members of a mock jury (Wheatley and Haidt 2005; Jones and Fitness 2008; Eskine et al. 2011; Brown et al. 2017; Salerno 2017; but cf. Landy and Goodwin 2015a, 2015b). Yet bear in mind that the propensity to feel emotions other than disgust—anger, sadness, fear—predicts stricter moral judgments too (Landy and Piazza 2019). Thus, severe moralizing might be a product of strong emotions in general, rather than of disgust in particular.

It is possible to bypass the use of repugnant pictures, smells, and descriptions, and manipulate disgust more directly. This can be done with ginger, a spice which is known to reduce nausea and has now been found to reduce modest levels of disgust as well (Tracy et al. 2019). The effect of ginger is small, but in this series of experiments, involving several hundred participants each, participants who had ingested capsules filled with ginger judged moderately revolting transgressions as less reprehensible than those who had ingested placebo capsules similar in taste, but filled with sugar.

Altogether, these findings suggest that the symbolism of idioms such as washing one’s hands of something (to refuse responsibility for it) is far more grounded than one might have thought. So is the significance of rituals like baptism, rinsing one’s feet before prayer, or taking a dip in the Ganges to clean one’s soul.

### Infection threat sways our political choices

Moral and religious convictions permeate political ones, and it has repeatedly been found that right-wing conservatives get disgusted more easily than do left-wing liberals (one such study had over 30,000 participants: Inbar et al. 2012; see also Terrizzi et al. 2013; Inbar and Pizarro 2016; but cf. Bakker et al. 2020). This seems the case at least for issues like abortion, homosexuality, and immigration (Terrizzi et al. 2010; Kim et al. 2016; Aarøe et al. 2017), though not nearly as much for problems such as fair taxation (Elad-Strenger et al. 2020). Yet of course, abortion and homosexuality are bound to invoke mental images of bodily fluids like blood and semen (and immigration, images of mass invasions) much sooner than economic issues do. The fact that women tend to be more collectivistic, conservative, and religious than men appears largely driven by their being more easily disgusted than men about sexual matters (Terrizzi et al. 2014).

How easily we become disgusted drives not only our political preferences but also our actual votes, in both mock and real elections (Inbar et al. 2012; see also Beall et al. 2016). People who have just viewed or read something disgusting prefer an attractive (hence, as we have seen, seemingly healthier) political candidate over an unattractive one more than do people who have viewed or read something else (White et al. 2013). Disgust for body odors predicts harsh moral attitudes and authoritarianism and, through the latter, support for right-wing US president Donald Trump (Liuzza et al. 2018, 2019). That odors might play a role in political preferences is also tentatively suggested by a genome-wide analysis of the DNA of over 13,000 people: one region of chromosome 9, which contains a large number of genes involved in the sense of smell, appears associated with variation in conservative-liberal political orientation (Hatemi et al. 2011).

At first sight, these findings are consistent with the idea that political conservatism and an authoritarian attitude might be behavioral immune responses to infection threats. Indeed, decreases in parasite prevalence predict later increases in democracy (Kusano and Kemmelmeier 2020). However, two independent studies suggest that activation of the behavioral immune system can push people’s views not only toward the right, as shown by many, but occasionally toward the left too (Feinberg and Willer 2013; Kam and Estes 2016; see also Elad-Strenger et al. 2020). One of these studies (Feinberg and Willer 2013) compared three messages. The first was about how important it is to clean the environment and showed disgusting pictures of a pollution cloud, a forest covered in garbage, and someone drinking contaminated water. The second was about how important it is to protect the environment and showed nondisgusting pictures of a destroyed forest, a barren coral reef, and cracked land. The third was a neutral message on the history of neckties. Among the participants who had read the disgust-inducing message (and only those), conservatives ended up supporting the typical left-wing issue of environmental care just as much as did liberals.

Whether issues such as abortion or the rights of homosexual individuals could also be conveyed so as to change minds one way or another—and not only in the laboratory but outside of it as well—remains to be seen. Still, the point is that political preferences and votes are swayed not just by rational arguments (if at all), but by a deliberate or accidental invocation of people’s concerns about infection, too.

## The barbarity of civilization

The real world is messier than a controlled study, but it is events in the former that our theories ultimately need to predict. As we shall see, moving away from a society of hunters-gatherers to a complex civilization eventually brought us better health and greater harmony. Yet civilizations can collapse, and have repeatedly done so. Not seldom, it was the trading in of long-term prospects for short-term gains that finished them off (Diamond 2005): and once again we appear to be tempting our fate.

### The mixed blessings of the agricultural revolution

Around 10,000 years ago, circumstances pushed our ancestors away from hunting, gathering, and small-scale cultivation, toward large-scale agriculture and animal husbandry (Diamond 1997; Scott 2017). These developments had two notable consequences. First, the quantity of available food rose enormously and with it did the number of people, who lived of course in close proximity both to one another and to livestock (Cohen 1989; Scott 2017). Second, the variety and quality of such food dwindled, as rather few plants proved domesticable and not all of these were ideal for human consumption (a problem which persists today: Bressan and Kramer 2016). Judging from the remains of bones, teeth, and mummified organs, malnutrition and illness became commoner and life shorter (Cohen 1989; Murphy 2007; Scott 2017). With animals and (sickly) humans crammed together, jumping from one species to another was for germs a breeze, and epidemics became more frequent (Diamond 1997; Scott 2017).

This deterioration of public health, however, was not accompanied by a rise in aggression—as one would expect from the mobilization of thousands of behavioral immune systems—but by a marked decline (Pinker 2011, 2018). For this apparent contradiction to the argument put forth in this review, one possible reason stands out. The food surpluses produced by civilization enabled the emergence of a ruling class which restricted the right to use violence to the affiliates that protected its power—which today, on behalf of government, are the police and the military (Diamond 1997; Pinker 2011, 2018; Scott 2017). This gradual monopolization of violence robbed those left out of it of the freedom to settle scores themselves: a coerced pacification which may have overshadowed any effects of worsening public health on people’s aggressive tendencies.

Overproduction paved the way for public works, including sanitation and hospitals. Throughout the twentieth century, in part thanks to the diffusion of vaccination and the introduction of antibiotics, public health did finally improve (Ward and Warren 2006): from 1950 to 2017, the global average life expectancy rose from 53 to 76 years for women and from 48 to 71 for men (GBD 2017 Mortality Collaborators 2018). At the same time, in line with the viewpoint presented here and despite the impression one gets from television, the odds of being discriminated, abused, tortured, or deliberately killed have sunk lower, and democracy and civil rights risen higher, than ever before (Pinker 2011, 2018; Kusano and Kemmelmeier 2020). Yet our current prospects may not be nearly as good as one might feel entitled to expect.

### Glimpses of the future

By disrupting the climate, polluting the environment, and replacing wilderness with farmland, roads, and cities, we invade other animals’ natural habitats or force them to invade ours. This mixing of humans and animals, escorted by their respective parasites, lends infections fresh opportunities to break out (Quammen 2012; Shah 2016; Wu et al. 2017; Johnson et al. 2020). In developing countries, the growth of farm animals is pushed with antibiotics, creating antibiotic resistance and making animal and human infections much harder to treat (Van Boeckel et al. 2019). In Europe and the USA, the tighter regulations on antibiotic use are of little help, possibly because animals crammed indoors get sick often and end up being given antibiotics anyway (Capita and Alonso-Calleja 2013). And at markets in East Asia, Africa, and elsewhere, all manner of wild animals—from bats to crocodiles—are amassed and even butchered together, a standing invitation to cross-infections and novel epidemics (Quammen 2012; Shah 2016; Wu et al. 2017).

Humans have always been gracious hosts to parasites and today they are even more so—as shown by the significant increase, over the period 1940–2004, in the number of infectious diseases emerging in human populations for the first time (Jones et al. 2008). Not only are we more densely packed, and traveling faster, than ever before: but in addition, the immune barriers pathogens need to elude are being torn down by pollution (Winans et al. 2011), drug misuse (Manchikanti and Singh 2008), and poor food choices (Kanneganti and Dixit 2012). In nations as advanced as the USA and the UK, on account of such developments, many can now expect to live only as long as their parents or grandparents, or less (GBD 2017 Mortality Collaborators 2018; Hiam et al. 2018; Woolf and Schoomaker 2019; Mehta et al. 2020).

It is not just actual threats to our health that are a worry. Following a potent evolutionary imperative—better safe than sorry—people prove more credulous than skeptical about potential hazards (Fessler et al. 2017); hence, they can feel even more vulnerable to infection than they actually are. Throughout the ages, all sorts of dark figures have profited from such gullibility, depicting minorities, enemies, and political opponents as carriers of germs or invading pests (Dunstan 2010; Tipler and Ruscher 2014; Utych 2018). Today’s public is manipulated far more efficiently by personalized fake news on social media such as Facebook (Stark 2018; Wylie 2019). Fake news can create false beliefs (Carey et al. 2020) and memories of events that never happened (Murphy et al. 2019). It also evokes more surprise, fear, and disgust than does news that one can double-check (Vosoughi et al. 2018). And once we become disgusted, we just want to look away (Armstrong et al. 2014; Oosterhoff et al. 2018) and avoid the disgusting matter, not find out more about it (Clifford 2018).

Alas, the spread of both actual and imaginary infections is bound to press on people’s behavioral immune systems. There are signs indeed—time will tell whether they mark a transitory aberration or a lasting change—that our millennia-long progress toward tolerance and harmony has stopped, with large sections of society turning more and more hostile toward immigrants, ethnic and religious minorities, intellectual “elites,” and other groups. Apparently, mainstream politics is now joining the trend (Carothers and O’Donohue 2019; Norris and Inglehart 2019).

## Infection and sociality: insights from an interdisciplinary view

This review has presented a particularly broad perspective on the relationship between sociality and infectious disease. It has brought together research on immunity, hormones, personality, sexual habits, cultural differences, the foundation of morality, political orientation and persuasion, social media and fake news, and the history of agriculture and animal domestication. The integration of such diverse material into one coherent narrative has the potential to produce fresh insights. As a case in point, behavioral responses to contagion threat turn out to be impressively similar to those that follow a sniff of oxytocin; inactivating the oxytocin receptor, or deleting its gene, even eliminates behavioral immune reactions in rodents. Yet when it comes to research on humans, those who study oxytocin appear to take little notice of the behavioral immune system (but see Declerck et al. 2010), and those who study the behavioral immune system little notice of oxytocin. Bridging the divide could help constrain theories in both fields.

Likewise, the literature on prejudice appears split between one line of research, reviewed here, that explicitly links prejudice to contagion risk, and another that does not consider the behavioral immune system at all (e.g., Pettigrew 2016). There is little communication between the two, but they could join forces in all kinds of interesting ways. For example, it has been shown that interacting physically, virtually, or even just in our imagination with people from another group, or watching a member of our own group do so, lessens our prejudices (Dovidio 2017). The roots of this phenomenon are unclear. Yet interacting with strangers makes them more familiar: and strangers whose faces seem more familiar inspire more comfort with contact, largely because they look healthier (Bressan 2020). This may go a long way towards explaining why engaging with new people lowers our biases against them. Indeed, the downside of familiarity is that it can induce an underestimation of the infection threat these individuals pose. The ensuing intimacy may do little damage when one faces pathogens that have been going around for a long time, since we are likely to have already been exposed to the parasites carried by familiar people—but could be catastrophic when the pathogen is novel to all, as in times of COVID-19 (Bressan 2020).

A final example of the advantages of an interdisciplinary view of the relationship between infection and sociality concerns the argument that prejudice against strangers is unrelated to the avoidance of unfamiliar parasites. Some have proposed that discrimination only applies to strangers one specifically associates—via socially transmitted information—with ecologies that are wracked with infectious diseases (Ji et al. 2019); or that the trouble with strangers is that they tend to violate local norms and traditions (Tybur et al. 2016) or are stigmatized by political entrepreneurs (van Leeuwen and Petersen 2018). Yet animals other than humans, like mice, have a behavioral immune system too and a bias against strangers too, despite most such animals being unlikely to consider their conspecifics’ respect of norms and traditions, to associate newcomers with specific ecologies, or to have political entrepreneurs in their midst. Evidence from animal research makes it implausible that human-centered explanations of discrimination go to the heart of the problem.

## Coda: the hidden returns of investing in public health

Keeping a broad perspective might be helpful not just for science but for society too. While COVID-19 has killed over 2 million people within its first year, the outbreak of the next lethal pandemic is merely a matter of time; a prime candidate for it has been identified already (Sun et al. 2020). Preventing pandemics is undoubtedly an expensive affair, requiring as it does programs to test livestock, curtail deforestation, reduce the opportunity for viruses to affect new hosts, monitor the trade of wildlife, and stop that of wild meats. Such interventions may cost up to 31 billion dollars per year (Dobson et al. 2020). Yet productivity losses due to COVID-19 are estimated to exceed 5 trillion dollars for 2020 alone—11.5 trillion in the end. Not considering their ancillary advantages, these preventive measures would already be cost-effective if they reduced the probability of another pandemic in the next year by only about one-fourth (Dobson et al. 2020). And of course, the evidence discussed in this review suggests that warding off infections would benefit us beyond that, even just from a purely economic stance. After all, productivity is bound to go up not only when people are healthier but also when they are more sociable and inclined to work with, rather than against, one another.

## Data Availability

No datasets were generated or analyzed in this study.
